# Divergent topological architecture of the default mode network as a pretreatment predictor of early antidepressant response in major depressive disorder

**DOI:** 10.1038/srep39243

**Published:** 2016-12-14

**Authors:** Zhenghua Hou, Zan Wang, Wenhao Jiang, Yingying Yin, Yingying Yue, Yuqun Zhang, Xiaopeng Song, Yonggui Yuan

**Affiliations:** 1 Department of Psychosomatics & Psychiatry, Institute of Psychosomatics, Zhongda Hospital, Medical School of Southeast University, Nanjing 210009, China; 2 Department of Psychiatry, Affiliated Wuhu NO.4 Hospital of Shanghai Jiaotong University BIO-X center, Wuhu 241001, China; 3 Department of Neurology, Institute of Neuropsychology, Zhongda Hospital, Medical School of Southeast University, Nanjing 210009, China; 4 Department of Biomedical Engineering, College of Engineering, Peking University, Beijing 100871, China

## Abstract

Identifying a robust pretreatment neuroimaging marker would be helpful for the selection of an optimal therapy for major depressive disorder (MDD). We recruited 82 MDD patients [n = 42 treatment-responsive depression (RD) and n = 40 non-responding depression (NRD)] and 50 healthy controls (HC) for this study. Based on the thresholded partial correlation matrices of 58 specific brain regions, a graph theory approach was applied to analyse the topological properties. When compared to HC, both RD and NRD patients exhibited a lower nodal degree (D_nodal_) in the left anterior cingulate gyrus; as for RD, the D_nodal_ of the left superior medial orbitofrontal gyrus was significantly reduced, but the right inferior orbitofrontal gyrus was increased (all *P* < 0.017, FDR corrected). Moreover, the nodal degree in the right dorsolateral superior frontal cortex (SFGdor) was significantly lower in RD than in NRD. Receiver operating characteristic curve analysis demonstrated that the λ and nodal degree in the right SFGdor exhibited a good ability to distinguish nonresponding patients from responsive patients, which could serve as a specific maker to predict an early response to antidepressants. The disrupted topological configurations in the present study extend the understanding of pretreatment neuroimaging predictors for antidepressant medication.

Depression is a common psychiatric disorder that accounts for the highest proportion of global burden attributable to mental disorders^1^. Major depressive disorder (MDD) is characterized by deep sadness, reduced energy, autonomic nerve dysfunction, cognitive dysfunction and even high suicidal tendency^2^. Although other treatment choices are available, antidepressant medication (ADM) treatments are the front-line options for MDD. Regarding clinical efficacy, only approximately 50% of patients respond to frontline antidepressants, and less than 33% obtain remission^3^. In current clinical practice, clinicians need more than 6–8 weeks to judge the primary outcome of an antidepressant according to the symptom changes. Recently, early symptom improvements have been identified as a valuable predictor of eventual outcome to ADMs^4^,^5^,^6^; however, the objective indexes that could anticipate the early response of drugs are still lacking. The identification of pretreatment predictors is clinically relevant given that an adequate understanding of a predictor for the response to antidepressant treatment can help reduce the public health burden and suicide risk. However, knowledge of the neurobiological mechanisms underlying discrepant antidepressant outcomes is still fragmented and incomplete.

We previously detected abnormal functional connectivity using resting-state functional magnetic resonance imaging (rs-fMRI) within the default mode network (DMN) and salience network (SN), including the posterior cingulate cortex^7^, hippocampus^8^, amygdala^9^ and cortico-cerebellar regions^10^. However, emerging evidence has changed the viewpoint that MDD is just related to aberrant activation or connectivity in sparse brain regions, but rather, it is now thought to consist of disconnected syndromes that undermine the function of large-scale brain networks subserving the emotional response to stress^11^,^12^,^13^. The uncovered imaging predictors of treatment response have not yet been effectively applied to clinical practice, and the implicit interconnections among distributed brain networks at the global level in MDD patients are still unclear. Graph theory is a mathematical method that enables us to investigate the topological pattern of complex brain networks by generating a matrix of the interconnected edge of nodes. However, the results concerning the disrupted topological properties in MDD were contradictory. Measuring the topological properties, Zhang *et al*.^12^ found imbalanced functional segregation and integration in first-episode MDD, that is, a lower path length and higher global efficiency of global functional networks, and aberrant nodal centrality of local networks. In contrast, another study revealed reduced global efficiency but increased global centrality in MDD^14^. Specifically, recent research has suggested that the abnormal anatomical topological patterns (deficit patterns of node strength) were related to antidepressant treatment in depressed patients^15^. To date, a study that focuses on the prognostic value of the topological feature of DMN to predict the early response to antidepressant therapy is warranted. These predictors from neuroimaging findings may prompt clinicians to more precisely choose effective treatment types for patients, providing a promising option for personalizing therapy.

In the present study, we hypothesize that the global topological patterns were disorganized and nodal efficiency of the small-world network was mainly changed in posterior regions of the default mode network. The main aim of this study was to explore potential changes in the topological architecture of the DMN in MDD patients with different early treatment responses. We also further investigated the specificity and sensitivity of these intrinsic topological alterations to differentiate the MDD patient who might show an early response to antidepressant treatment.

## Methods and Materials

### Participants

The Southeast University Research Ethics Committee approved the study in accordance with the Declaration of Helsinki, and written informed consent was obtained from all participants. All participants were recruited from the Affiliated Zhongda Hospital of Southeast University, China. All subjects were interviewed in a semi-structured interview included in the Structured Clinical Interview for DSM-IV Axis I Disorders (SCID-I/P), Clinician Version. To avoid misdiagnoses, the baseline diagnoses of MDD were determined by another senior psychiatrist in the follow-up period. All participants (patients and healthy controls (HC)) also underwent diagnostic evaluations including a clinical interview and the Hamilton depression rating scale (HAMD), review of medical history and demographic inventory. The detailed inclusion and exclusion criteria are described in Supplemental Materials. All subjects were unequivocally and naturally right-handed. During the 2-week follow-up period, 5 patients refused to participate in the study. The treatment for the MDD group was as follows: 49 patients used selective serotonin reuptake inhibitor (SSRIs); 26 patients used serotonin-norepinephrine reuptake inhibitor (SNRIs); and 7 patients received an agglomeration of antidepressant combinations (SSRIs, SNRIs or mirtazapine). According to the reduction rate [defined as RR = (HAMD_baseline_−HAMD_2nd-week_)/HAMD_baseline_] of HAMD scores^16^, the MDD group was further subdivided into a non-responding depression (NRD, n = 40) (RR ≤ 50%) and responsive depression group (RD, n = 42) (RR > 50%).

### MRI acquisition

All subjects underwent the MRI scans at the Affiliated Zhongda Hospital of Southeast University. The subjects were scanned using a Siemens 3.0 Tesla scanner with a homogeneous birdcage head coil. Subjects lay supine with their head snugly fixed by a belt and foam pads to minimize head motion. The MRI acquisition sequences generated 240 volumes in 8 minutes and 176 slices in 4.3 minutes. All subjects were instructed to keep their eyes closed, relax, remain awake and to not think anything specific during scanning.

### MRI data preprocessing

Functional images were preprocessed utilizing the Data Processing Assistant for Resting-State fMRI (DPARSF 2.3) toolkit^17^, which synthesizes procedures based on the Resting-State Functional MR imaging toolkit (REST; http://www.restfmri.net)^18^ and the statistical parametric mapping software package (SPM8 http://www.fil.ion.ucl.ac.uk/spm). The key procedures included the removal of the first ten volumes, correction for timing differences and motion effects, spatial normalization to the Montreal Neurological Institute (MNI) space, linear detrending, temporal band-pass filtering (0.01–0.08 Hz), and regression of nuisance signals involving 6 head motion parameter corrections (participants with head motion of more than 1.5 mm of maximum displacement in any direction (x, y, or z) or 1.5 degrees of angular motion were excluded from the present study), as well as acquiring the global mean signal, cerebrospinal fluid signal and white matter signal (details see Supplemental Materials).

### Functional network construction

To construct the brain functional network of the DMN, in the present study, we focused on the DMN and selected a set of 58 regions of interest (ROIs) for DMN parcellation (see Supplementary Table 1). To measure interregional resting-state functional connectivity, Pearson correlation coefficients between any pair of ROIs were calculated, thus generating a 58 × 58 correlation matrix for each subject. Each absolute correlation matrix was then thresholded into a binary matrix with a fixed sparsity level, S (defined as the number of edges in a graph divided by the maximum possible number of edges in the graph). As there is no golden standard for a single threshold, we thresholded each absolute correlation matrix repeatedly over a relatively wide range of sparsity levels (6% ≤ *S* ≤ 34%) at an interval of 0.01 and calculated the parameters of the resulting graphs with different thresholds. The details of the network construction can be found in Supplemental Materials.

### Network Analysis

For the constructed brain networks at each sparsity threshold, we calculated both global and regional network measures. The global measures included (1) small-world parameters^19^ involving clustering coefficient (C_p_), characteristic path length (L_p_), normalized clustering coefficient (γ), normalized characteristic path length (λ) and small-worldness (σ); and (2) network efficiency^20^ involving local efficiency (E_loc_) and global efficiency (E_glob_). To determine the nodal (or regional) characteristics of the brain networks, we computed the nodal efficiency (E_nodal_) and degree (D_nodal_)^21^. For a recent review on the applications and interpretations of these network measures, see the research from Rubinov *et al*.^22^ and Supplemental Materials. Moreover, we also calculated the area under the curve (AUC)^12^ for each network metric, which provides a summarized scalar for topological characterization of brain networks independent of the single threshold selection.

### Statistical Analysis

Independent sample t-test, Chi-square test, analysis of covariance (ANCOVA) and Bonferroni post hoc test (SPSS17.0, Chicago) were used to determine significant differences in demographic data, HAMD scores, characteristics of depression and topological measures among the three groups. Subsequent between-group analyses of topological measures were determined by ANCOVA with age, gender and education level as covariates. Furthermore, a false discovery rate (FDR)^23^ correction was applied for multiple comparisons of the regional graph theoretical calculations. The altered regions are shown on the surface of the brain using the BrainNet Viewer software^24^ (http://www.nitrc.org/projects/bnv). The continuous variables are presented as the mean ± SD. Receiver operator characteristic (ROC) curve analysis was applied to determine the optimal threshold for distinguishing NRD and RD [area under curve, (AUC): 0.9–1 = excellent; 0.8–0.9 = good; 0.7–0.8 = fair; 0.6–0.7 = poor; 0.5–0.6 = fail]. To further validate the synergetic distinguishing effect of those topological features, the binary logistic regression analysis was applied to generate the predictive values of each potential index, and then the ROC analysis was able to merge these predictive values to get a combined ROC curve. We defined statistical significance at *P* < 0.05.

## Results

### Demographic and pathological characteristics

No significant differences in age were observed (F = 0.41, *P* = 0.67); however, the gender distribution (χ^2^ = 8.52, *P* = 0.01) and education levels (F = 5.91, *P* < 0.01) were significantly different among the three groups. Post hoc multiple comparisons showed that education levels of both the NRD (mean difference I-J = −2.34, *P* = 0.03) and RD group (mean difference I-J = −2.84, *P* = 0.01) were significantly lower than the HC; no significant differences of disease duration (t = 0.761, *P* = 0.45), baseline HAMD score (t = 0.043, *P* = 0.97), gender (χ^2^ = 0.02, *P* = 1.00) or education level (mean difference I-J = 0.50, *P* = 1.00) were detected between NRD and RD (Table 1). After the multiple correction of FDR (*P* < 0.017), the gender difference was non-significant in the RD (χ^2^ = 6.32, *P* = 0.02) and NRD (χ^2^ = 5.51, *P* = 0.03) groups compared to that of the HC. The effects of age, gender and education were controlled in the following statistical analyses.

### Topological properties of functional brain networks

**Figure 1:**
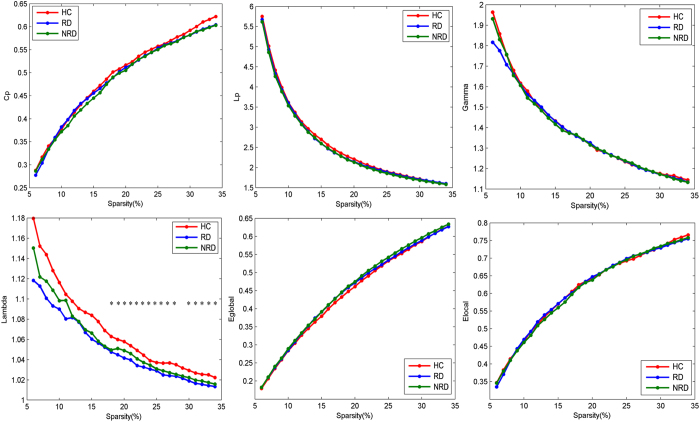
The graphs show small-world parameters and network efficiency of the DMN. Clustering coefficient (**A**), characteristic path length (**B**), normalized clustering coefficient (**C**), normalized characteristic path length (**D**), global efficiency (**E**) and local efficiency (**F**) of the RD (blue line) and NRD (green line) groups and the NC (red line) as a function of sparsity thresholds. Black asterisks (*) indicate the significant difference between groups (*P* < 0.05). Abbreviations: DMN, default mode network; RD, responsive depression; NRD, nonresponding depression; NC, Normal Controls.

Over the entire range S of 0.06–0.34, the functional brain networks of RD, NRD and HC exhibited greater clustering coefficients than random networks but almost equivalent characteristic path lengths; those results represent a typical topology characteristic of small-worldness. Although sharing common small-world organizations, further analyses uncovered significant differences in small-world parameters. No significant intergroup differences were detected in the *Cp, Lp*, γ, or global and local efficiency (E_glob_ & E_loc_) over the whole range of sparsity. However, the results indicated significant differences of λ among the three groups at a wide range of sparsity (18% ≤ S ≤ 28%, 30% ≤ S ≤ 34%). Comparing the AUC values of the normalized path lengths, the results revealed that the λ of both the RD (F = 11.18, *P* = 0.001) and NRD (F = 5.59, *P* = 0.02) groups were prominently lower than that of the HC (Fig. 1); there was no significant difference in the λ value between the RD and NRD groups (F = 0.93, *P* = 0.34).

### Alterations in regional nodal characteristics

**Figure 2:**
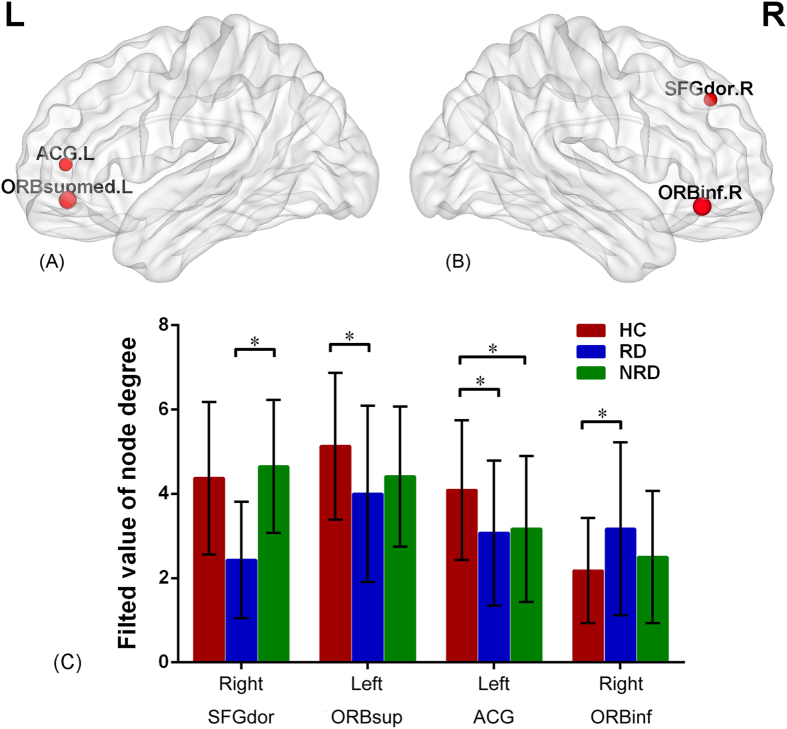
The brain areas exhibit different nodal degrees among the three groups (subfigure (**A,B**). The inset bar plot indicates the between-group differences of RD and NRD in the AUC values of each nodal degree (based on Bonferroni post hoc test). Abbreviations: DMN, default mode network; RD, responsive depression; NRD, nonresponding depression; AUC, area under the curve; SFGdor, dorsolateral superior frontal gyrus; ORBsup/inf, orbitofrontal gyrus of superior/inferior part; ACG, anterior cingulate gyrus; L, left; R, right. The picture is shown on the surface of the brain using the BrainNet Viewer software (http://www.nitrc.org/projects/bnv).

When compared to HC, both the RD and NRD groups exhibited a lower nodal degree (D_nodal_) in the left anterior cingulate gyrus. The D_nodal_ of the RD group was significantly smaller in the left superior medial orbitofrontal gyrus (ORB_supmed_) but bigger in the right inferior orbitofrontal gyrus (ORB_inf_) (all *P* < 0.017, FDR corrected) than that of the HC. Moreover, the nodal degree in the right dorsolateral superior frontal cortex (SFGdor) was dramatically smaller in the RD group than in the NRD group (*P* < 0.017, FDR corrected) (Fig. 2). No significant differences in nodal efficiency were observed among the three groups.

### The performance of topological network properties in the differentiation of NRD from RD

**Figure 3:**
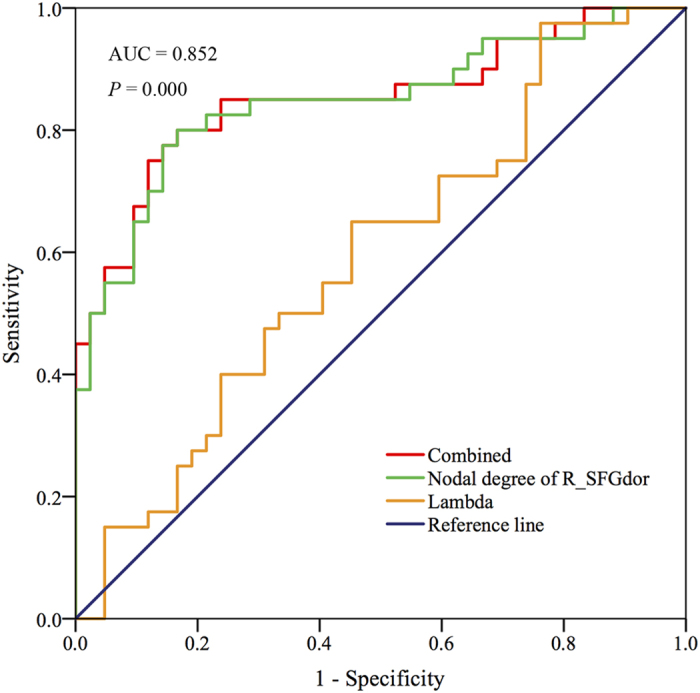
ROC curves based on the binary logistic regression model using the combination of the altered nodal degree and lambda to distinguish NRD from RD. Abbreviations: ROC, receiver operating curve; NRD, nonresponding depression; RD, responsive depression.

As detailed in Table 2, ROC curve analysis indicated that the AUC of the nodal degree in the right SFGdor was 0.847 [95% confidence interval (CI): 0.76–0.93, *P* < 0.001] with a sensitivity of 78% and specificity of 86%. When the lambda and nodal degree of the right SFGdor are further combined into the ROC analysis, the AUC was optimized to 0.852 (95% CI: 0.77–0.94, *P* < 0.001) with a sensitivity of 80% and specificity of 83%, indicating that these two measures could serve as potential diagnostic predictors to differentiate the NRD group from the RD group (Fig. 3).

### Relationships between network parameters and HAMD score

**Figure 4:**
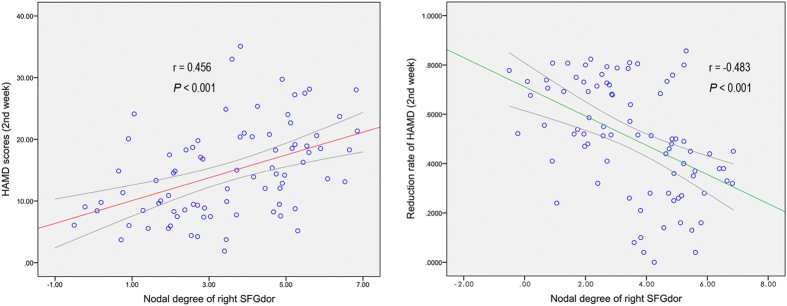
Partial correlation analysis showed that the nodal degree of the right SFGdor was significantly positively correlated with the HAMD score (2nd week) but negatively correlated with the reduction rate of HAMD (2nd week) in the pooled MDD group, adjusted for age, sex and education. Abbreviations: HAMD, Hamilton depression rating scale; SFGdor, dorsolateral superior frontal gyrus; MDD, major depressive disorder.

After adjusting for age, sex and education, the partial correlation analysis showed that the nodal degree of the right SFGdor was significantly positively correlated with the HAMD score at week 2 (r = 0.456, *P* < 0.001, corrected with Bonferroni, *P* < 0.005) but negatively correlated with the reduction rate of the HAMD (r = −0.483, *P* < 0.001, corrected with Bonferroni, *P* < 0.005) in the pooled MDD group (RD plus NRD) (Fig. 4). There were no significant correlations found between the global or nodal network measures and pre- or post-treatment HAMD scores in the RD or NRD group.

## Discussion

This study was designed as a pragmatic investigation for the identification of pretreatment imaging predictors of early antidepressant response using the topological architecture of brain functional networks in MDD. Our major results were as follows: 1) MDD patients exhibited significantly altered global topological properties of brain functional networks as characterized by aberrant normalized short path lengths; 2) different nodal characteristics (nodal degree) were found mainly in orbitofrontal-limbic regions, including a reduced D_nodal_ of the left ACG in both RD and NRD groups relative to HC and a decreased D_nodal_ of the left ORB_supmed_ but increased D_nodal_ of the right ORB_inf_ in the RD group compared to HC; 3) the NRD group showed a higher nodal degree in the right SFGdor than the RD group. Notably, ROC analysis substantiated the normalized short path length (λ), and the D_nodal_ in the right SFGdor can discriminate the NRD patients from the RD group. These results strengthen the credibility that the topological architecture of the brain function connectome is disrupted in MDD and provide preliminary evidence for the understanding of the neuropathological mechanism involved in the early response to antidepressant medication.

### Altered small-world measures in RD and NRD

As an optimal and economical network system, the human brain presents efficient small-world organizations that ensure a strong balance between global integration and local specialization for information processing^25^,^26^. The present study revealed that the functional brain topology of RD, NRD and HC exhibited economical, small-world features. These results were supported by previous evidence from a graph theory study in MDD^12^,^27^. Despite similar brain topological attributes, the global network metrics, characterized by the normalized path length, were significantly smaller in RD and NRD than in healthy controls. Generally, the measure of the path length represents the connection of distant brain regions that underlie long-distance information propagation^28^. The lower path length could enhance the global efficiency of information processing in the DMN, which is commonly overactivated in the process of maladaptive rumination with attentional bias to negative emotion stimuli in MDD^29^. In the present study, the topology with intensive global integration and stable regional efficiency reflect the disruption of the optimal balance in the brain network and exhibit a shift toward randomized organization, which has been observed in many neuropsychological diseases, including MDD^12^,^30^,^31^. By extension, the altered small-world configuration in MDD may be related to the self-referential processes underpinned by the DMN and may then provide the extended perspective that the neuronal network organization was remarkably disrupted in MDD.

### Disrupted nodal degree in the functional brain networks

As a typical measure of nodal centrality, nodal degree, represents the importance of a brain region in the local specialization. Our results suggested that both RD and NRD showed lower nodal degree in the left ACG than healthy controls. The ACG is a key brain region implicated in the emotional and cognitive process^32^ and is responsible for the top-down modulation of attention to internal and external stimuli and for the formation of corresponding affective reactions^33^. A morphological study uncovered a dramatic reduction in white matter and grey matter in the anterior cingulate in MDD^32^. In addition, an fMRI study also revealed that the depression-related hypoactivity in the anterior cingulate cortex^34^ could be reversed by effective antidepressant treatment^35^. Importantly, a recent positron emission tomography study further demonstrated that patients with effective antidepressant treatment displayed higher activity in the anterior cingulate cortex^36^. We propose that the disrupted local centrality in the ACC may negatively affect emotion regulation in the pathogenesis of MDD.

Simultaneously, we also confirmed that the altered nodal degrees in RD were concentrated in the orbitofrontal areas, which showed a reduced D_nodal_ in the left orbitofrontal superior part but an enhanced D_nodal_ in the right orbitofrontal inferior part compared to that in controls. The DMN is a constellation of brain areas with relatively separate anatomy, connections, and functionality. The ORB regions are critical DMN structures for self-focusing during confrontation of emotion stress^33^. A recent fMRI study^37^ demonstrated that depressed subjects exhibited pretreatment increased connectivity in the orbitofrontal cortex and the dorsomedial prefrontal cortex, which was also significantly correlated with the white matter burden. Importantly, a recent study detected lower activity in the left but higher activity in the right orbitofrontal cortex in response to aversive stimuli^38^. The discrepancy of the nodal centrality in bilateral ORB regions implies a functional imbalance in processing distinct emotional reactions. In the present study, the lower nodal degree in the left ORB suggested a weaker function of segregation in the transfer of emotional information. In addition, this dampened pattern of nodal centrality in RD rather than in NRD indicated that attenuation in the recruitment of the left ORB reflected a greater ability to compensate for MDD-related impairment and to thereby exert a facilitated response to treatment^39^. Future studies integrating modified designs would help to elucidate the confusion of different parts of the ORB (i.e., superior and inferior) in the modulation of emotional experience.

In the present study, the D_nodal_ in the right dorsolateral superior frontal gyrus was significantly positively correlated with the HAMD score at week 2 but negatively correlated with the reduction rate of HAMD in the pooled MDD group. Meanwhile, the RD patients displayed a smaller D_nodal_ in the right SFGdor than in the NRD group, which also exhibited a significant power to differentiate the NRD patients from the RD group. The SFGdor is a core structure of the DMN subserving introspective emotions and cognitive functions^40^. Neuroimaging studies have demonstrated that depressed patients show higher activation in the SFG^41^ and increased functional connectivity between the dorsolateral and medial superior frontal cortex^42^. This study uncovered distinct patterns of neural substrates underlying emotional regulation, which were correlated with the different responses to antidepressant treatment between RD and NRD. We cautiously speculate that the disrupted function of the DMN (i.e., heightened nodal degree of the SFGdor) in NRD partly causes the dysfunction of the cognitive control network^33^,^43^, which could mediate top-down regulation of the negative emotion to the external world^11^. Our results partly confirmed and extended the understanding that the maladaptive regulation of brain networks is related to the treatment response of MDD^44^. Even though treatment-responsive and nonresponsive MDD patients share overlapping depression symptoms, there are also distinct neural network underpinnings for different treatment responses.

Several limitations warrant attention and suggest directions for future research. First, due to the study design, which did not include an MRI scan at the 2-week follow-up, whether these regions with aberrant topological properties are altered with antidepressant treatment needs to be demonstrated. Further longitudinal studies are warranted to detect more sensitive and robust neural biomarkers for early response to antidepressants. In addition, utilizing optimized analytical strategies and multiple imaging modalities to explore the structural substrate underlying the drug-related topological abnormality would be beneficial. Secondly, similar to the previous study on antidepressant efficacy and prediction^45^, different antidepressants were prescribed in the present study. Further recruitment of patients using the same antidepressant would contribute to determining whether the findings of this study change according to antidepressant treatment. Finally, the present three groups were not well matched for education, although the confounder of education was removed in all the network analyses. However, these results should be interpreted with caution.

Taken together and building on previous work, the present study suggests that the analysis of the topological architecture may provide an informative perspective to examine the neuro-substrate of early treatment response in MDD. However, further investigation of the potential pathophysiological mechanism of topology alteration in MDD is warranted. In summary, the disrupted topological configurations in the present study extend the nascent understanding of the exploration of pretreatment neuroimaging predictors of early response to antidepressants therapies.

## Additional Information

**How to cite this article**: Hou, Z. *et al*. Divergent topological architecture of the default mode network as a pretreatment predictor of early antidepressant response in major depressive disorder. *Sci. Rep.* **6**, 39243; doi: 10.1038/srep39243 (2016).

**Publisher’s note:** Springer Nature remains neutral with regard to jurisdictional claims in published maps and institutional affiliations.

## Supplementary Material

Supplementary Materials

## Figures and Tables

**Table 1:** Demographic and neuropsychological characteristics of all groups.

Group	NC (n = 50) Mean ± SD	NRD (n = 40) Mean ± SD	RD (n = 42) Mean ± SD	Test statistic	*P* value
Age, years	46.48 ± 17.65	46.95 ± 15.45	49.31 ± 13.49	F_2,129_ = 0.41	0.67
Education level, years	11.92 ± 4.33	9.53 ± 4.20	9.02 ± 4.25	F_2,129_ = 5.91	< 0.01*
Gender, male/female	26/24	11/29	11/31	χ^2^ = 8.52, df = 2	0.01^#^
Baseline HAMD	NA	29.78 ± 5.86	29.71 ± 6.84	t = 0.04, df = 80	0.97
Duration, months	NA	6.30 ± 8.48	5.00 ± 7.06	t = 0.76, df = 80	0.45
Handedness, right/left	50/0	40/0	42/0	NA	NA

For comparisons of demographics: *P values were obtained using one-way ANOVA tests; ^#^*P* value for the gender distribution among the three groups was obtained using a Chi-square test. *P* < 0.05 was considered significant. Abbreviations: NC, normal controls; NRD, nonresponding depressed group at 2nd week; RD, responsive depressed group at 2nd week; HAMD, Hamilton depression rating scale; NA, not applicable. Parametric values are represented as the mean ± SD (standard deviation).

**Table 2:** The ROC of combined indexes for differentiating the NRD from the RD group.

Parameter	AUC	*P*	95%CI	Sensitivity	Specificity	Youden’J	Cut-point
Lambda	0.593	0.145	0.47–0.72	0.98	0.24	0.22	0.29
Nodal degree of R_ SFGdor	0.847	<0.001	0.76–0.93	0.78	0.86	0.64	0.59
Combined index	0.851	<0.001	0.77–0.94	0.80	0.83	0.63	3.65

The AUC of the combined index was acquired from the integrated predictive effects of the above two parameters. Abbreviation: ROC, receiver operator characteristic; NRD, non-responding depression; RD, responsive depression; AUC, area under curve; 95% CI, 95% confidence interval; R, right; SFGdor, dorsolateral superior frontal cortex.
