# Supplementary material for: Divergent topological architecture of the default mode network as a pretreatment predictor of early antidepressant response in major depressive disorder
Source: Sci Rep. 2016 Dec 14;6:39243. doi: 10.1038/srep39243 (PMC5155246; doi:10.1038/srep39243)
Supplement: Supplementary Materials [file srep39243-s1.pdf]

Divergent topological architectures of default mode network as pretreatment predictors of early antidepressants response in major depressive disorder

Zhenghua Hou[1, 4], Zan Wang[2], Wenhao Jiang[1], Yingying Yin[1], Yingying Yue[1], Yuqun Zhang[1], Xiaopeng Song[3] & Yonggui Yuan[1]

[1]Department of Psychosomatics & Psychiatry, Institute of Psychosomatic Medicine, Zhongda Hospital, Medical School of Southeast University, Nanjing 210009, China

[2]Department of Neurology, Institute of Neuropsychology, Zhongda Hospital, Medical School of Southeast University, Nanjing 210009, China

[3]Department of Biomedical Engineering, College of Engineering, Peking University, Beijing 100871, China.

[4]Department of Psychiatry, Affiliated Wuhu NO.4 Hospital of Shanghai Jiaotong University BIO-X center, Wuhu 241001, China

Correspondence and requests for materials should be addressed to Yonggui Yuan. (Email: yygylh2000@sina.com)

Supplementary Materials

Supplemental Method

Participants

The MDD participants met the following inclusion and exclusion criteria: (1) they met the major depressive disorder in DSM-IV criteria at the time point of enrollment; (2) they were in their first depressive episode and the age of onset was over 18 years; (3) 24 items Hamilton Depression Rating Scale (HAMD) were greater than 20; (4) absence of another major psychiatric illness, including severe anxiety, substance abuse or dependence; (5) absence of primary neurological illness, including dementia or stroke; (6) absence of medical illness impairing cognitive function; (7) no history of receiving electroconvulsive therapy; (8) no gross structural abnormalities on T1-weight images, and no major gross major white matter changes such as infarction or other vascular lesions T2-weighted MRI; (9) have no psychotic symptoms (i.e. hallucination/bizarre delusions/thought broadcasting). For healthy controls (HC) that recruited from local community, they must meet the abovementioned criteria (4) - (9) and have no history of any affective disorders including MDD.

Imaging Acquisition

A gradient-recalled echo-planar imaging (GRE-EPI) pulse sequence was set up to acquire resting-state images. The acquisition parameters of rs-fMRI were as follows: repetition time = 2000 ms; echo time = 25 ms; flip angle = 90°; acquisition matrix = 64 × 64; field of view = 240 × 240 mm^2; thickness = 3.0 mm; gap = 0 mm; 36 axial slices, and 3.75 × 3.75 mm^2 in-plane resolution parallel to the anterior commissure-posterior commissure line. High-resolution T1-weighted axial images covering the whole brain were acquired utilizing a 3-dimensional inversion recovery prepared fast spoiled gradient echo (SPGR) sequence presented as follows: repetition time = 1900ms; echo time

= 2.48ms; flip angle = 9° ; acquisition matrix = 256 × 192; field of view = 250mm × 250mm; thickness =1.0mm; gap = 0mm. The MRI scans were processed before the patients get start to receive antidepressants treatment. After the removed of head motion (i.e., exceeding 1.5 mm in transition or 1.5° in rotation) or poor quality of image (i.e., ghost intensity), the MRI data from 82 MDD patients and 50 HC qualify for further calculate.

Functional Image Preprocessing

Functional images were preprocessed utilizing the Data Processing Assistant for Resting-State Function MRI (DPARSF 2.3 http://www.restfmri.net/forum/dparsf) toolkit, which synthesizes procedures based on the Resting-State Functional MR imaging toolkit (REST; http://www.restfmri.net), and statistical parametric mapping software package (SPM8 http://www.fil.ion.ucl.ac.uk/spm). The first ten time points were discounted in order to ensure stable-state longitudinal magnetization and adaptation to inherent scanner noise. The remaining 230 rs-fMRI images were sequentially performed according following steps: (1) slice timed with the 35th slice as reference slice; corrected for temporal differences and head motion correction (participants with head motion of more than 1.5 mm of maximum displacement in any direction (x, y, or z) or 1.5 degrees of angular motion were excluded from the present study); (2) coregistered T1 to functional image and then reoriented; (3) for spatial normalization, T1-weighted anatomic images were segmented into white matter, gray matter and cerebrospinal fluid, and then normalized to the Montreal Neurological Institute space by using a 12-parameter nonlinear transformation. The above transformation parameters were applied to the functional images and then the functional images with isotropic voxels of 3 mm resampled; (3) spatial smoothing undertaken with a 6 mm full-width at half-maximum isotropic Gaussian kernel; (4) the linear trend within each voxel's time series

removed; (5) temporal bandpass (0.01-0.08Hz) to minimize low-frequency drift and high-frequency noise filtered; (6) the nuisance signals (global mean signal, white matter, cerebrospinal fluid signals, head-motion parameters calculated by rigid body 6 correction) and spike regressors were regressed out.

Network Construction

A whole-brain parcellation scheme was recently created based on a large meta-analysis of fMRI studies combined with whole brain functional connectivity mapping [1]. This set of 264 putative functional regions was demonstrated more effective to construct the accurate information of network (i.e., optimize the detection of previously addressed functional networks such as salience networks, and DMN) than voxel-wise and atlas-based parcellation approaches. Therefore, in the present study, we focused on the DMN and selected a set of 58 regions of interest (ROIs) for DMN parcellation (Supplementary eTable 1). The subgraph (i.e., DMN) derived using these ROIs show substantial agreement with task-dependent functional neural system defined previously [1]. For each subject, we further calculate the Pearson's correlation coefficients between the mean time-courses of all possible pairs of 58 ROIs, generating a 58 × 58 correlation matrix. To avoid complicated statistical descriptions in the following network analysis, our graph theoretical analysis is confined to a simple undirected and unweighted binary matrix (Supplementary eFigure 1). Each absolute correlation matrix was thresholded into a binary matrix with a fixed sparsity level, S (defined as the number of edges in a graph divided by the maximum possible number of edges of the graph). Setting a sparsity threshold ensured that all the resultant networks had the same number of edges [2,3].

Network Analysis

Small-world Parameters

The small-world parameters of a network (clustering coefficient *Cp*, and characteristic path length *Lp*) were originally proposed by Watts and Strogatz [4]. Briefly, the *Cp* of a network is the average of the clustering coefficients over all nodes, where the clustering coefficient *Ci* of a node is defined as the ratio of the number of existing connections among the node's neighbors and all their possible connections. *Cp* quantifies the local interconnectivity of a network. *Lp* of a network is the shortest path length (numbers of edges) required to transfer from one node to another averaged over all pairs of nodes. *Lp* indicates the overall routing efficiency of a network. To estimate the small-world properties, we scaled *Cp* and *Lp* derived from the brain networks with the mean Cp^{rand} and Lp^{rand} of 100 random networks (i.e., $\gamma = Cp/ Cp^{rand}$ and $\lambda = Lp/ Lp^{rand}$) that preserved the same number of nodes, edges and degree distributions as the real networks [5]. A small-world network should fulfill the conditions of $\gamma > 1$ and $\lambda \approx 1$ [4], and then the small-worldness scalar $\sigma = \gamma / \lambda$ will be higher than 1.

Network Efficiency

The global efficiency measures the ability of parallel information transmission over the network [6]. For a network G with N nodes and K edges, the global efficiency of G can be computed as:

$$E_{glob}(G) = \frac{1}{N(N-1)} \sum_{i \neq j \in G} \frac{1}{L_{ij}},$$

where L_{ij} is the shortest path length between node *i* and node *j* in G.

The local efficiency measures the fault tolerance of the network, indicating the capability of information exchange for each subgraph when the index node is eliminated. The local efficiency of G is measured as:

$$E_{loc}(G) = \frac{1}{N} \sum_{i \in G} E_{glob}(G_i),$$

where *Gi* denotes the subgraph composed of the nearest neighbors of node *i*.

Regional Nodal Characteristics

To evaluate the roles of brain regions (or nodes) in brain networks, we computed the regional efficiency $E_{nodal}(i)$ [2]. Nodal efficiency measures the information propagation ability of a node with the rest of nodes in the network. The nodal efficiency of node *i* is computed as:

$$E_{nodal}(i) = \frac{1}{N(N-1)} \sum_{i \neq j \in G} \frac{1}{L_{ij}},$$

where L_{ij} is the shortest path length between node *i* and node *j* in *G*.

Finally, the nodal degree of a node *i* is defined as:

$$D_{nodal} = \sum_{j \neq i \in G} e_{ij},$$

where e_{ij} is the (i,j) element in the formerly generated binary, undirected network.

Statistical Analysis

Network metrics

To test the null hypothesis that the observed group differences could occur by chance, we randomly reallocated each subject to one of the two groups and recomputed the mean differences between the two randomized groups. The randomization procedure was repeated 10,000 times, and a randomized null distribution based on between-group differences in each metric was created. Then the 95% percentile point of the distribution was used as the critical value for two-tail test of the null hypothesis. This permutation test procedure was repeated at the sparsity of 6% $\leq$ S $\leq$ 34%. Additionally, the same permutation procedure was used to analyse the AUC of network measures between groups. Furthermore, before the permutation tests, multiple linear regression analyses were applied to regress the confounding effects of age, gender and years of education for each network metric.

Supplementary eTable 1. The Cortical and Subcortical Regions of Interest Defined in the Study

Index	Brain Region	Abbreviation	Index	Brain Region	Abbreviation
01	Left middle occipital gyrus	MOG.L	30	Right superior frontal gyrus, medial	SFGmed.R
02	Right superior frontal gyrus, orbital part	ORBsup.R	31	Right superior frontal gyrus, medial	SFGmed.R
03	Right gyrus rectus	REC.R	32	Left anterior cingulate	ACG.L
04	Left lingual gyrus	LING.L	33	Right superior frontal gyrus, medial	SFGmed.R
05	Left superior frontal gyrus, orbital part	ORBsup.L	34	Left superior frontal gyrus, medial orbital part	ORBsupmed.L
06	Left middle temporal gyrus	MTG.L	35	Right superior frontal gyrus, medial orbital part	ORBsupmed.R
07	Right middle occipital gyrus	MOG.R	36	Left anterior cingulate	ACG.L
08	Left temporal pole: middle temporal gyrus	TPOmid.L	37	Left superior frontal gyrus, medial	SFGmed.L
09	Right temporal pole: middle temporal gyrus	TPOmid.R	38	Left anterior cingulate and paracingulate gyri	ACG.L
10	Left inferior temporal gyrus	ITG.L	39	Left superior frontal gyrus, dorsolateral	SFGdor.L
11	Left angular gyrus	ANG.L	40	Left superior frontal gyrus, medial	SFGmed.L
12	Left parietal, but supra-marginal and angular gyrus	IPL.L	41	Right middle temporal gyrus	MTG.R
13	Left precuneus	PCUN.L	42	Left middle temporal gyrus	MTG.L
14	Right precuneus	PCUN.R	43	Left middle temporal gyrus	MTG.L
15	Left precuneus	PCUN.L	44	Right middle temporal gyrus	MTG.R
16	Left retrosplenial complex	RSC.L	45	Left middle temporal gyrus	MTG.L
17	Posterior cingulate gyrus	PCG.R	46	Right superior frontal gyrus, dorsolateral	SFGdor.R
18	Right precuneus	PCUN.R	47	Right anterior cingulate and paracingulate gyri	ACG.R
19	Median cingulate and paracingulate gyri	DCG.L	48	Right middle temporal gyrus	MTG.R
20	Right precuneus	PCUN.R	49	Left parahippocampal gyrus	PHG.L

Index	Brain Region	Abbreviation	Index	Brain Region	Abbreviation
21	Right angular gyrus	ANG.R	50	Right fusiform gyrus	FFG.R
22	Right superior frontal gyrus, dorsolateral	SFGdor.R	51	Left fusiform gyrus	FFG.L
23	Left superior frontal gyrus, medial	SFGmed.L	52	Right cerebellum crus, area 1	Cerebellum_Crus1_R
24	Left superior frontal gyrus, dorsolateral	SFGdor.L	53	Right temporal pole: middle temporal gyrus	TPOmid.R
25	Left middle frontal gyrus	MFG.L	54	Left middle temporal gyrus	MTG.L
26	Right superior frontal gyrus, dorsolateral	SFGdor.R	55	Right angular gyrus	ANG.R
27	Right superior frontal gyrus, dorsolateral	SFGdor.R	56	Middle temporal gyrus	MTG.L
28	Left superior frontal gyrus, dorsolateral	SFGdor.L	57	Left inferior frontal gyrus, orbital part	ORBinf.L
29	Left superior frontal gyrus, dorsolateral	SFGdor.L	58	Right inferior frontal gyrus, orbital part	ORBinf.R

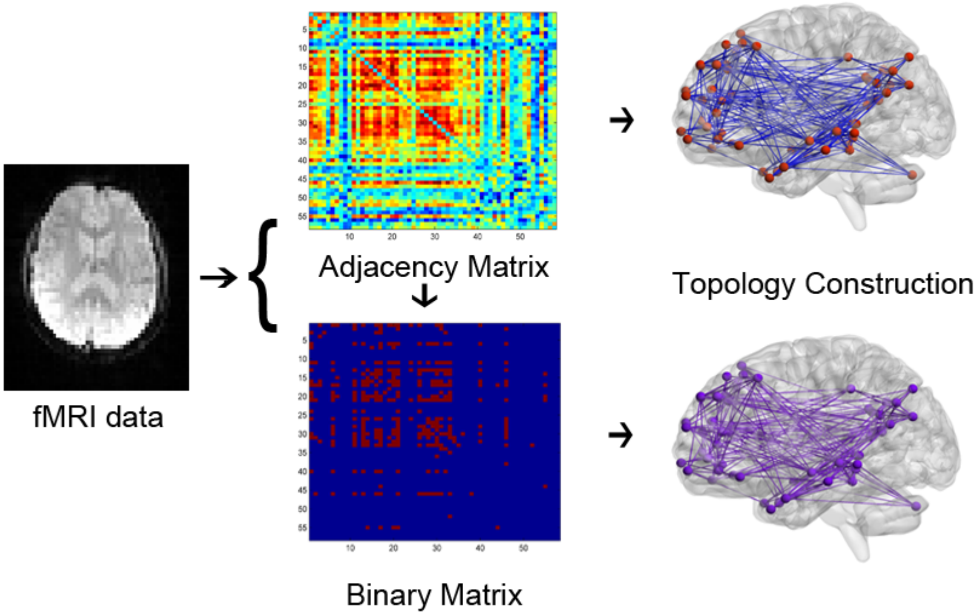

Supplementary eFigure 1. A flowchart for the construction of functional brain networks.

Note: For all subjects, a correlation matrix was acquired for each subject by calculating inter-regional Pearson's correlation coefficient of mean time series among the 58 DMN regions and can generate the non-binarized topological matrices; then, these correlation matrices were further converted into binarized matrices by applying a thresholding procedure; finally, the obtained binary matrices could be finally represented as networks or graphs that were composed of brain nodes and edges.

References

1. Power, J. D. *et al.* Functional network organization of the human brain. *Neuron.* **72**, 665-678 (2011).

2. Achard, S. & Bullmore, E. Efficiency and cost of economical brain functional networks. *PLoS Comput Biol.* **3**, e17 (2007).

3. Zhang, J. *et al.* Disrupted brain connectivity networks in drug-naïve, first-episode major depressive disorder. *Biol psychiatry.* **70**, 334-342 (2011).

4. Watts, D. J. & Strogatz, S. H. Collective dynamics of 'small-world' networks. *Nature.* **393**, 440-442 (1998).

5. Maslov, S. & Sneppen, K. Specificity and stability in topology of protein networks. *Science.* **296**, 910-913 (2002).

6. Latora, V. & Marchiori, M. Efficient behavior of small-world networks. *Phys Rev Lett.* **87** (2001).
